# Should Nurses Smoke?

**Published:** 1921-03-12

**Authors:** 


### SHOULD NURSES SMOKE?

The smoking habit, which exercises the minds of many, is usually and improperly regarded as appertaining to the domain of conduct. In the initial stage there may be some ground for this, as in the case of boys; but this aspect of smoking soon vanishes, as in the case of men. Old-fashioned people look askance at women who smoke, and they advance all kinds of threadbare arguments why they should not, and so unreasonable and illogical do these appear that they have quite the opposite effect to that intended. They simply stir up a spirit of antagonism and revolt at a code of ethics devised in the early Victorian era and in certain circles still regarded as sacred. Personally, I regard smoking as a pernicious habit for the great majority of women, and certainly for nurses. But I do not think it is by any means a question of ethics.

### Forging the Chain.

Have you ever considered why a young man begins to smoke? In a vulgar monosyllable, the answer is—swank. As soon as he finds himself in long trousers he feels that he has cast off the child and put on the man, and it becomes immediately apparent to him that he cannot smoke, so he hides himself away, does the necessary penance, and emerges, in his own opinion at all events, a man. In the eyes of older people he is a young fool. The old veteran recognises that in this effort to grow old before his time the lad is stunting his growth and, maybe, undermining his constitution.

Despite the subtle charm that has been woven round My Lady Nicotine by worshipping multitudes, it is beyond dispute that the effect of smoking is to reduce vitality and gratuitously to impose a tax on the nervous system. Those who enjoy tobacco most are young men in their prime, whose occupation is out of doors, and who on this account can afford to indulge without suffering harm. Men who lead sedentary lives know that they are better without smoking, and they are continually making spasmodic and vain efforts to abandon the habit. Sometimes they are pleased with themselves when they succeed in making a substantial reduction in their daily consumption. They abstain for Lent, more as a hygienic than as a religious exercise, and they know that the tobacco habit is a chain on their freedom which their will power is too weak to break. Smoking, in fact, is not all pure joy. I do not think there is any exception to the rule that one must pay for one's narcotics, and while in the case of tobacco, to vigorous men with an apparently unlimited supply of nervous energy, the price may appear to be trivial, it is a different matter in the case of boys who have not finished growing, and likewise of girls who have to work hard and to whom physical fitness is a most important concern.

### Cigarettes.

Smokers agree that the cigarette is the least wholesome smoke. I have heard various reasons ascribed, including the fact that paper is an essential part. But I think the fact is that there is a tendency with cigarette smokers to inhale, and there can be no doubt that inhaling is most damaging to the health. Inhalers have no desire for any other form of smoking. The most delicious of cigars makes no appeal, the fact being that the inhaled fumes of the cigarette more nearly approach a drug than any other use of tobacco.

It is extremely difficult to improve on Nature, or to bamboozle Nature. Am I to be told that smoking is restful?—that it soothes the nerves? The same applies to all opiates, and to this and every such there is an aftermath. What of sleep?

> "that knits up the ravelled sleeve of care,
> The death of each day's life, sore labour's bath,
> Balm of hurt minds, great Nature's second course,
> Chief nourisher in life's feast."

The present consumption of cigarettes in this country is appalling. It is calculated to bring about national decadence. The Legislature recognise this in the case of boys by making it a penal offence in the Children Act. Having passed the age of childhood, people are left to themselves to carve out their destinies, with here and there an exception, as in the case of cocaine.

### Ask the Veterans.

Personally, I believe that smoking for young women is damaging to their health. It saps their energy, and causes them to look old before their time.

> "It taints the breath, the blood it dries,
> It burns the head, it blinds the eyes,
> It dries the lungs, scourgeth the lights,
> It numbs the soul, it dulls the sprites."

If they would decide whether to smoke or not to smoke, I suggest that they should, before they begin, ask one or two men who were smokers of experience, old chaps who may be relied upon to give honest advice. The subject is, admittedly, highly debatable, because the habit is so general that advocates are to be found without number amongst the devotees. Very few men, comparatively speaking, have the courage to lay bare their souls for the inspection of others. Rather do they endeavour to conceal the truth from themselves. It is this frankness that makes De Quincey's "Confessions" fascinating. I commend this little volume to every reader who is interested in the mystery of human nature. If, having read it, a young man or woman ventures to forge a chain which by any possibility may bring about even a mild form of slavery, then there is no more to be said.

A nurse, I hold, occupies a peculiar public position, one whose value is considerably underrated. Her duties are strenuous and responsible, and an essential factor to their success is physical fitness. What is called brightness in a nurse is an invaluable asset. I put it to the advocate of nicotine, does smoking tend in any respect to add to her pleasure, or to her physical, mental, or moral well-being?

With its conformity to the conventions I have no concern.

IERNE.

